# High levels of uric acid inhibit BAT thermogenic capacity through regulation of AMPK

**DOI:** 10.1152/ajpendo.00092.2023

**Published:** 2023-09-21

**Authors:** Meijuan Dong, Kun An, Li Mao

**Affiliations:** ^1^Department of Endocrinology, The Affiliated Huaian No.1 People’s Hospital of Nanjing Medical University, Huaian, China; ^2^Department of Neurology, The Affiliated Huaian No.1 People’s Hospital of Nanjing Medical University, Huaian, China

**Keywords:** AMPK, brown fat, mitochondria, thermogenesis, uric acid

## Abstract

Hyperuricemia (HUA) is strongly associated with the increasing prevalence of obesity, but the underlying mechanism remains elusive. Dysfunction of brown adipose tissue (BAT) could lead to obesity. However, studies on the role of HUA on BAT are lacking. Our retrospective clinical analysis showed that serum uric acid (UA) is significantly associated with BAT in humans. To investigate the role of UA in regulating BAT function, we used UA to treat primary brown adipocytes (BACs) in vitro and established HUA mice. In vitro results showed that HUA suppressed thermogenic gene expression and oxygen consumption rate. Accordingly, HUA mice exhibited lower energy expenditure and body temperature, with larger lipid droplets and lower thermogenic gene expression. These results demonstrate that HUA inhibits BAT thermogenic capacity in vitro and in vivo. To further elucidate the mechanism of UA on adipocytes, mRNA-sequencing analysis was performed and screened for “AMP-activated protein kinase (AMPK) signaling pathway” and “mitochondrial biogenesis.” Further tests in vivo and in vitro showed that the phosphorylation of AMPK was suppressed by HUA. Activation of AMPK alleviated the inhibition of AMPK phosphorylation by HUA and increased mitochondrial biogenesis, subsequently restoring the impaired BAT thermogenic capacity in vitro and vivo. Thus, we confirmed that HUA suppresses mitochondrial biogenesis by regulating AMPK, thereby inhibiting BAT thermogenic capacity. Taken together, our study identifies UA as a novel regulator of BAT thermogenic capacity, providing a new strategy to combat obesity.

**NEW & NOTEWORTHY** To investigate the effect and mechanism of UA on BAT thermogenic capacity, we established HUA models in vitro and in vivo, and performed RNA sequencing analysis. Our results revealed that HUA suppresses mitochondrial biogenesis by regulating AMPK, thereby inhibiting BAT thermogenic capacity. Taken together, our study identifies UA as a novel regulator of BAT thermogenic capacity, providing a new strategy to combat obesity.

## Graphical abstract

**Figure:**
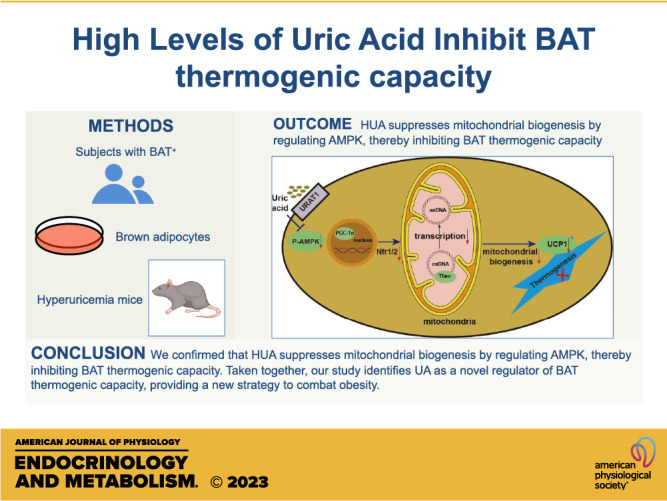

## INTRODUCTION

Hyperuricemia (HUA), an independent risk factor for central obesity (1), often occurs in patients with metabolic disorders, such as type 2 diabetes and hyperlipidemia (1–3). Diet-induced HUA mice exhibited obesity, insulin resistance, and non-alcoholic fatty liver (2, 4–6), suggesting that HUA is involved in the development of obesity. Obesity occurs when energy intake exceeds energy expenditure (7, 8), so increasing energy expenditure is considered a promising target for obesity prevention.

Adipose tissue is classified as white adipose tissue (WAT) and brown adipose tissue (BAT). BAT is mainly localized in cervical, supraclavicular, axillary, paraspinal, and perirenal depots. Unlike WAT, which stores energy in triglycerides, BAT dissipates energy by producing heat to maintain body temperature in a process known as nonshivering thermogenesis (9, 10). Brown adipocytes (BAC) are enriched in mitochondria whose inner membrane harbors uncoupling protein1 (UCP1), which uncouples oxidative respiration from ATP synthesis (11–13), leading to heat production. BAT can be recruited and activated by cold exposure or β-adrenoceptor stimulation (11). In humans, BAT was previously thought to disappear after birth. However, accumulating evidence from ^18^Fluorine-fluorodeoxyglucose (^18^F-FDG) PET scans suggests the existence of functional BAT in adult humans (7, 14). A recent retrospective study showed that BAT was independently correlated with a lower risk of type 2 diabetes, dyslipidemia, cardiovascular disease, and cerebrovascular disease (14). Therefore, promoting the thermogenetic capacity of BAT is considered a potential strategy to combat obesity.

The expression of *UCP1*, PPARc co-activator 1α (*PGC1*α), and PR domain containing 16 (*PRDM16*) in 3T3-L1 adipocytes induced by β-adrenergic agonist was attenuated by uric acid (UA) (4), indicating that UA may also regulate these thermogenetic genes in BAT. However, there is still a lack of relevant research and the mechanism remains elusive. UA is the end product of purine metabolism and is mainly transported by urate transporter-1 (URAT1), which is predominantly expressed in the kidney (15, 16). Recently, URAT1 has also been found to be expressed in BAT and WAT (4, 5, 16). Intracellular UA levels were significantly increased when adipocytes were exposed to a high concentration of UA, demonstrating that extracellular UA is transported into adipocytes via URAT1 (5, 16).

To investigate the effect and major mechanism of UA on BAT thermogenic capacity, we established HUA models in vitro and in vivo, and performed RNA sequencing analysis. Our data demonstrated that UA inhibits BAT thermogenic capacity through the regulation of AMP-activated protein kinase (AMPK). The results of this study identify UA as a novel regulator of BAT thermogenic capacity, providing a new strategy to combat obesity.

## METHODS

### PET-CT

Positron emission tomography-computed tomography (PET-CT) images from Huaian No.1 People′s Hospital from 2013 to 2022 were retrospectively analyzed and processed using Advantage Workstation AW 4.3-05 and MSviewer software to determine the presence of BAT and then calculate the volume and activity of BAT. Those who met both of the following two conditions were considered as BAT positive (17, 18): (1) The diameter of the ^18^F-FDG concentrated area range is more than 4 mm (mainly located in the neck, supraclavicular region, axillary and paraspinous areas) and the CT values range from −250 to −50 HU (the CT value range of the adipose tissue). (2) The standard uptake value (SUV) of the ^18^F-FDG concentrated area is more than 2.0. BAT activity was calculated according to the following formula: BAT activity = BAT volume (mL) × fat density (0.9 g/mL) × SUVmean. Subjects without tumor were divided into two groups according to whether BAT was positive or not. A total of 98 subjects were included in the BAT-positive group and 246 subjects in the BAT-negative group. Clinical and biochemical data were collected. The study was approved by the Ethics Committee of Huaian No.1 People’s Hospital, and the requirement for patient informed consent was waived.

### Cell Culture and Differentiation

Primary BAT stromal vascular cells (SVFs) were isolated from the interscapular BAT of 4-wk-old male C57BL/6 mice. Adipose tissue was minced and subjected to collagenase digestion (Sigma-Aldrich) to obtain BAT SVFs which were maintained in DMEM (Gibco) containing 10% FBS (Gibco) and penicillin (Gibco) and streptomycin (Gibco), according to our previous report (19). To induce differentiation of BAT SVFs, confluent cells (*day 0*) were exposed to a differentiation cocktail containing 20 nmol/L insulin (Sigma-Aldrich, St. Louis) and 1 nmol/L T3 (Gibco). After 8 days of induction, the BAT SVFs finally reached full maturation. Subsequently, mature brown adipocytes (BACs) were incubated for 24 h with or without the following reagents: UA (4 mg/mL, 8 mg/mL, or 12 mg/mL, Gibco), 1 nM CL-316,243 (CL, Tocris Bioscience, UK), 10 μM benzbromarone (Ben, Chemlin Chemical, Nanjing, China), 2 mM metformin (Met, Chemlin Chemical, Nanjing, China), 500 μM 5-aminoimidazole-4-carboxamide ribonucleotide (AICAR, Biolang Biotechnology, Shanghai, China) or 10 μM compound C (CC, Sigma-Aldrich, St. Louis).

### Animals

Six-week-old male C57BL/6J mice were purchased from Shanghai Laboratory Animal Center Laboratory Animal Co., Ltd. (Shanghai, China). Hyperuricemia was induced in mice according to Yong’s method (20). Briefly, mice received the treatment of daily intraperitoneal injection of potassium oxonate (PO, Aladdin Biochemical Technology, Shanghai, China) 300 mg/kg and oral gavage of hypoxanthine (HX, Aladdin Biochemical Technology, Shanghai, China) 500 mg/kg for seven consecutive days, while the control group received the same volume of saline. The mice were then randomly divided into four groups of seven mice each: normal control (NC), hyperuricemia (HUA), NC drug treatment, and HUA drug treatment. Mice in the drug treatment groups were given 20 mg/kg Ben or 300 mg/kg Met by gavage or 0.5 mg/g AICAR or 10 mg/kg CC by intraperitoneal injection (Sigma-Aldrich, St. Louis) for 4 wk. The same volume of saline was used in the NC and HUA groups. Meanwhile, hyperuricemic mice continued treatment with PO and HX for 4 wk.

To acutely activate BAT, mice were either injected subcutaneously with 1 mg/kg body weight of CL-316,243 or placed in a cold (6°C) environment 6 h before the experiments, according to Heine’s method (21). Body temperature was recorded for half an hour with a rectal probe connected to a digital thermometer. Indirect calorimetry was determined according to the following method. After temperature and indirect calorimetry measurements, all mice were anesthetized and then sacrificed to obtain whole blood samples and brown adipose tissue, which were stored at −80°C for further analysis. All procedures involving animals were performed in accordance with the guidelines of the Nanjing Medical University Committee on the Care and Use of Animals.

### Immunohistochemistry Staining and H&E Staining

Adipose tissues fixed in 4% paraformaldehyde were sectioned after paraffin embedding. Immunohistochemistry staining was performed according to the standard protocol using UCP1 antibody (Abcam, UK), incubated overnight in a humidified chamber at 4°C. Sections were then incubated with secondary antibody (Abcam, UK) at 37°C for 30 min. For H&E, tissues were fixed in 10% formalin and embedded in paraffin. Sections were then stained with hematoxylin and eosin (H&E). Microscopic examinations were performed and photographs were taken under a regular light microscope.

### Mitochondrial Respiration Assay

To determine the mitochondrial respiration activities, the O_2_ concentration in the cells was measured using an XF24 extracellular flux analyzer (Seahorse Bioscience North Billerica, MA). Briefly, preadipocytes were seeded into 24-well cell culture microplate (Seahorse Bioscience) and differentiated into brown adipocytes. Oxygen consumption rate (OCR) and extracellular acidification were measured in XF assay medium supplemented with 1 mM GlutaMax-1 (Gibco), 1 mM sodium pyruvate (Sigma-Aldrich), and 25 mM glucose. After baseline measurements, the following three sequential injections were applied to the cells as indicated: 1 μM oligomycin (Sigma-Aldrich), 1 μM carbonyl cyanide 4-trifluoromethoxy phenylhydrazone (FCCP, Sigma-Aldrich), and 1 μM rotenone (Sigma-Aldrich). Basal, uncoupled, and maximal OCR were calculated by averaging the values from each phase.

### Mitochondrial Content Measurement

Total DNA was isolated using the E.Z.N.A. Tissue DNA Kit (Omega Bio-tek, Norcross, GA), according to the manufacturer’s instructions. Mitochondrial content was evaluated by means of the ratio of the mitochondrial DNA (mtDNA) copy number to nuclear DNA (nDNA) copy number, quantified by qPCR, assuming that the nDNA copy number remained constant. For each DNA extract, the nuclear-encoded gene, ribosomal protein large p0 (*Rplp0*, ntDNA), and the mitochondria-encoded gene, cytochrome b (*Cytb*, mtDNA), were individually quantified by qPCR. Data were normalized to *Rplp0* using the 2−ΔΔCt method.

### mRNA Sequencing Analysis

Cutadapt software (https://cutadapt.readthedocs.io/en/stable/) was used to remove reads containing adaptor contamination. And after removing the low-quality bases and undetermined bases, we used HISAT2 software (https://daehwankimlab.github.io/hisat2/) to map the reads to the genome. The mapped reads from each sample were assembled using StringTie (http://ccb.jhu.edu/software/stringtie/) with default parameters. All transcriptomes from all samples were then merged to reconstruct a comprehensive transcriptome using gffcompare software (http://ccb.jhu.edu/software/stringtie/gffcompare.shtml). Once the final transcriptome was generated, the expression levels of all transcripts were estimated using StringTie and ballgown (http://www.bioconductor.org/packages/release/bioc/html/ballgown.html), and the expression levels of mRNAs were calculated using FPKM (Fragments Per Kilobase of exon model per Million mapped reads). The differentially expressed mRNAs with fold change > 2 or fold change < 0.5 and *P* value < 0.05 were selected by R package edgeR (https://bioconductor.org/packages/release/bioc/html/edgeR.html) or DESeq2 (http://www.bioconductor.org/packages/release/bioc/html/DESeq2.html), and then GO (Gene Ontology) KEGG (Kyoto Encyclopedia of Genes and Genomes) enrichment were analyzed on the differentially expressed mRNAs.

### RNA Isolation and Real-Time RT-PCR

Total RNA was extracted from cells or tissues using TRIzol reagent (Invitrogen). cDNA was reverse transcribed (Bio-Rad) and amplified with SYBR Green Supermix (Bio-Rad) using a LightCycler 480 Real-time PCR system (Roche). The mRNA levels of the designated genes were normalized to *β-actin*. The primer sequences used are described in Table 1 and Supplemental Table S1.

**Table 1.** List of primer sets for RT-PCR

Gene	NCBI Accession	Forward (5′ to 3′)	Reverse (5′ to 3′)
UCP1	NM_009463.3	ACTGCCACACCTCCAGTCATT	CTTTGCCTCACTCAGGATTGG
PRDM16	NM_001291029.1	CAGCACGGTGAAGCCATTC	GCGTGCATCCGCTTGTG
Cidea	NM_007702.2	ATCACAACTGGCCTGGTTACG	TACTACCCGGTGTCCATTTCT
Cox8b	NM_007751.3	CCAGCCAAAACTCCCACTT	GAACCATGAAGCCAACGAC
Tfam	NM_009360.4	TGAGGCAGTTTAGACAGAATGG	GGTTGAAGCCATGAGTTCTCTA
Nrf1	NM_0011B226.1	GGTGAAATAAGCCTCCCGATAG	TGAGGCAGGTTTAGACAGAATGG
Nrf2	NM_010902.5	CAGCATAGAGCAGGACATGGAG	GAACAGCGGTAGTATCAGCCAG
PGC-1α	NM_008904.2	GAAAGGGCCAAACAGAGAGA	GTAAATCACACGGCGCTCTT
β-Actin	NM_007393.5	CAACGAGCG GTTCCGATG	GCCACAGGATTCCA TACCCA

RT-PCR, reverse transcription-polymerase chain reaction.

### Western Blotting

To examine protein expression, cells or tissues were lysed in radioimmunoprecipitation assay lysis buffer (Beyotime, Shanghai, China) containing a protease inhibitor cocktail (Roche). Protein concentrations were determined using a standard bicinchoninic acid assay. The same number of protein samples were separated on 10% sodium dodecyl sulfate-polyacrylamide gels and transferred to polyvinylidene difluoride membranes (Bio-Rad). The membranes were incubated overnight at 4°C with one of the following primary antibodies: β-actin (Abcam, UK), UCP1 (Abcam, UK), p-AMPKα (Thr172), and AMPKα (Abcam, UK), followed by incubation with horseradish peroxidase-conjugated secondary antibodies (Abcam, UK). Signals were detected using ChemiDOCTMXRS+ and the Image Lab system (Bio-Rad).

### Assessment of Indirect Calorimetry

To study energy expenditure, mice were injected subcutaneously with 1 mg/kg body weight of CL316,243, and food was removed for the following 6 h. Indirect calorimetry was performed using a comprehensive laboratory animal monitoring system (CLAMS; Columbus Instruments). Metabolic parameters of oxygen consumption and carbon dioxide production were calculated throughout the experimental period. Energy expenditure was calculated using O_2_ consumption, CO_2_ consumption, and body mass. All data were analyzed using CalR (version 1.3, https://calrapp.org/), a web-based analysis tool for Indirect Calorimetry Experiments CalR (22).

### Measurement of UA Concentration

Serum UA levels were determined using the UA assay kit (Sigma). The concentration of UA in the supernatant was determined using the UA assay kit (Sigma), according to the manufacturer’s instructions. Intracellular UA levels of adipocytes were corrected based on the protein concentration of the supernatant determined by the Bradford protein assay.

### Statistical Analysis

Statistical analysis was performed using SPSS 22.0 software. Normality of distributions was assessed using the Kolmogorov–Smirnov test. Continuous variables with a normal distribution were presented as means ± SE, whereas variables with a skewed distribution were log-transformed before analysis. Two-tailed t-tests were used for comparisons between two groups, and one-way ANOVA for comparisons between more than two groups. For categorical variables presented as numbers and percentages, differences between groups were analyzed using the chi-square test. Variables that might affect BAT activity or BAT volume were analyzed using Pearson correlation and multiple linear regression. If the variance inflation factors (VIF) were less than 10, it was assumed that there was no collinearity between the variables included in the multivariate linear regression. A *P* value <0.05 was considered statistically significant.

## RESULTS

### Serum UA Is Significantly Associated with BAT Activity and BAT Volume in Humans

**Figure 1.**
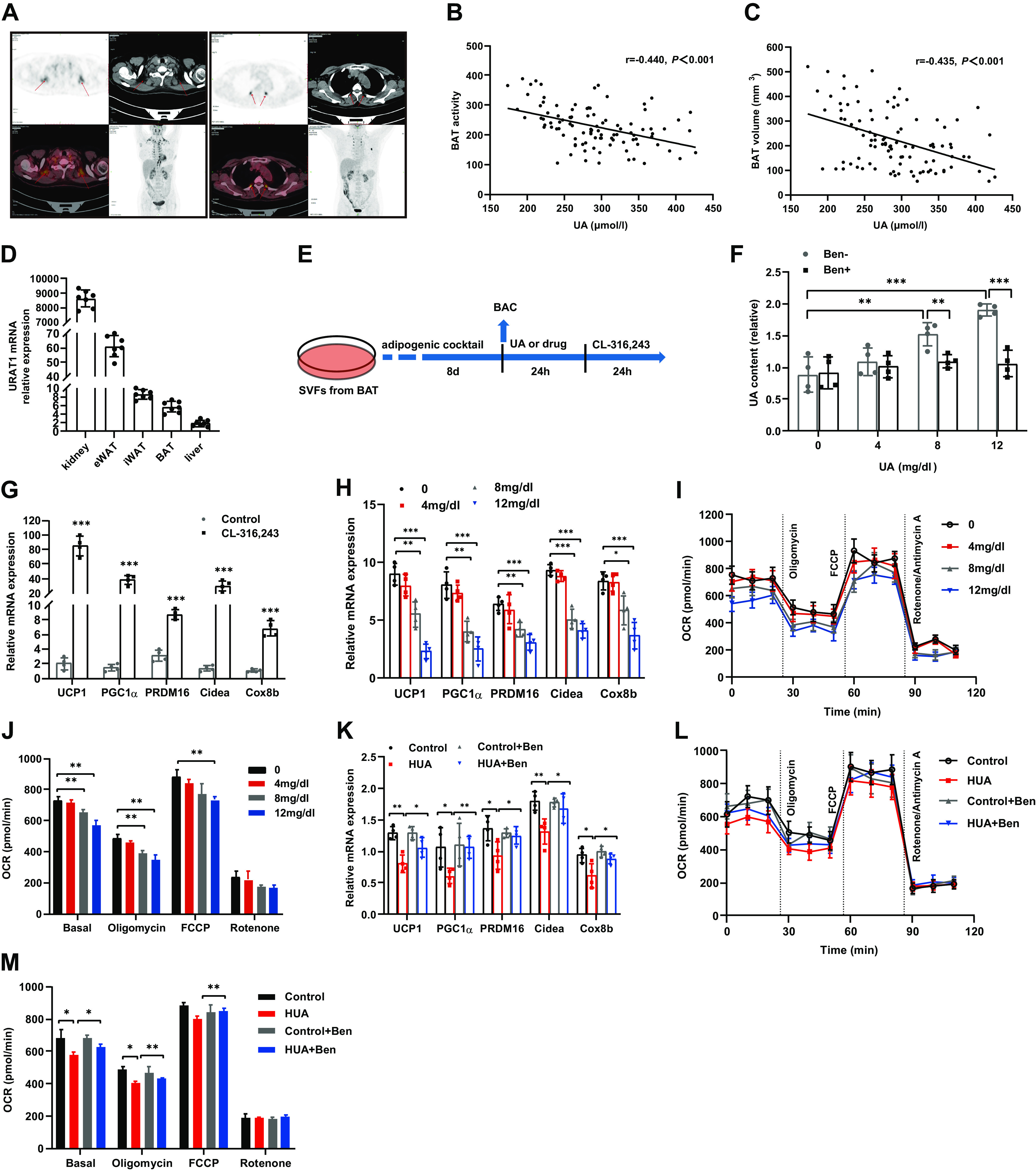
High levels of uric acid inhibit BAC thermogenic capacity in vitro. *A*: representative PET-CT images of BAT in humans: arrows indicate cervical (*left*) and paraspinal (*right*) BAT, respectively. *B*: Pearson correlation between BAT activity and uric acid (UA) in humans (*n* = 98). *C*: Pearson correlation between BAT volume and UA in humans (*n* = 98). *D*: relative mRNA expression of URAT1 in kidney, eWAT, iWAT, BAT, and liver measured by qRT-PCR (*n* = 7). *E*: flowchart of brown adipocyte (BAC) differentiation and UA treatment, drug refers to benzbromarone (or metformin, AICAR and Compound C in the latter figure). *F*: the levels of intracellular UA (*n* = 4). *G*: mRNA expression of thermogenic genes in the BAC treated with the β3-adrenergic receptor activator agonist CL-316,243 measured by qRT-PCR (*n* = 4). *H*–*M*: BAC were treated with CL-316,243 to activate the thermogenic capacity before measured. *H*: mRNA expression of thermogenic genes in the BAC treated with different concentrations of UA measured by qPCR (*n* = 4). *I*: oxygen consumption rate (OCR) of the BAC treated with different concentrations of UA, measured with an XF24 extracellular flux analyzer (*n* = 4). *J*: the average OCR of the BAC described in (*I*) at different stages. *K*–*M*: the HUA model of BAC was established by treatment with 8 mg/dL UA, as the average serum uric acid concentration in HUA mice was 8 mg/dL (Fig. 2*B*). *K*: mRNA expression of thermogenic genes in BAC from different treatment groups measured by qRT-PCR (*n* = 4). *L*: oxygen consumption rate (OCR) of the BAC from different treatment groups measured with an XF24 extracellular flux analyzer (*n* = 4). *M*: the average OCR of the BAC described in (*L*) at different stages. Data are shown as means ± SE. Statistical significance by Student’s *t* test or one-way ANOVA (*P < 0.05, **P < 0.01, ***P < 0.001). *n* represents the number of subjects, mice or cell samples in each group. URAT1, urate transporter-1; Ben, benzbromarone treatment; UCP1, uncoupling protein1; PGC1α, peroxisome proliferator-activated receptor γ co-activator 1α; PRDM16, PR domain containing 16; Cidea, cell death-inducing D FFA-like effector A; Cox8b, cytochrome *c* oxidase 8b; FCCP, carbonyl cyanide 4-trifluoromethoxy phenylhydrazone.

To study the potential association between UA and BAT in humans, we retrospectively analyzed PET-CT images and biochemical data of 344 cases and divided the 344 cases into BAT-positive group (*n* = 98) and BAT-negative group (*n* = 246). Representative PET-CT images of BAT were shown in Fig. 1*A*. The results showed that body mass index (BMI), UA, fasting blood glucose (FBG), triglyceride (TG), and low-density lipoprotein cholesterol (LDL-C) in the BAT-positive group were lower, while high-density lipoprotein cholesterol (HDL-C) was higher than those in the BAT-negative group (Table 2). According to the correlation analysis in the BAT-positive group, BAT activity was negatively correlated with BMI (*r* = −0.521, *P* < 0.001), UA (*r* = −0.440, *P* < 0.001), FBG (*r* = −0.291, *P* = 0.004) and TG (*r* = −0.225, *P* = 0.001) (Fig. 1*B* and Supplemental Fig. S1, *A*–*C*), and BAT volume was also negatively correlated with BMI (*r* = −0.464, *P* < 0.001), UA (*r* = −0.435, *P* < 0.001), FBG (*r* = −0.371, *P* < 0.001), and TG (*r* = −0.331, *P* = 0.001) (Fig. 1*C* and Supplemental Fig. S1, *D*–*F*). Multiple linear regression analysis was further performed on the factors that may affect BAT volume and BAT activity (age, gender, UA, BMI, FBG, and TG) with BAT activity and BAT volume as dependent variables, respectively. No significant statistical collinearity was observed among the six variables. The analysis showed that UA and BMI were significantly associated with BAT activity (*F* = 9.152, *P* < 0.001, adjusted *R*^2^ = 0.316, Supplemental Table S2) and BAT volume (*F* = 8.474, *P* < 0.001, adjusted *R*^2^ = 0.316, Supplemental Table S3) after adjusting for age, gender, FBG, and TG.

**Table 2.** Characteristics of BAT positive and BAT negative subjects

Variables	BAT^+^ Group (*n* = 98)	BAT^−^ Group (*n* = 246)	*P* Value
Age, yr	55.62 ± 18.2	58.11 ± 15.58	0.170
Male, *n* (%)	46 (46.9)	140 (56.9)	0.119
Smoking, *n* (%)	20 (20.4)	54 (22.0)	0.884
Drinking, *n* (%)	25 (25.5)	54 (22.0)	0.481
Hypertension, *n* (%)	14 (14.3)	40 (16.3)	0.744
Diabetes, *n* (%)	9 (9.2)	21 (8.5)	0.834
Hyperlipidemia, *n* (%)	17 (17.3)	54 (22.0)	0.379
SBP, mmHg	118.23 ± 11.59	116.02 ± 12.28	0.126
DBP, mmHg	69.11 ± 8.26	70.75 ± 9.43	0.133
BMI, kg/m^2^	24.32 ± 2.11	24.84 ± 2.27	0.049
ALT, U/L	22.69 ± 13.40	23.36 ± 16.61	0.723
AST, U/L	24.41 ± 9.36	22.54 ± 9.90	0.110
UA, μmol/L	290.29 ± 57.46	326.18 ± 106.36	0.002
BUN, mmol/L	5.23 ± 1.15	5.20 ± 1.55	0.853
Cr, μmol/L	65.93 ± 13.70	67.39 ± 15.11	0.403
FBG, mmol/L	4.77 ± 0.68	5.28 ± 1.50	0.001
TG, mmol/L	1.53 ± 0.67	1.94 ± 1.33	0.004
TC, mmol/L	4.46 ± 0.99	4.51 ± 0.84	0.606
LDL-C, mmol/L	2.54 ± 0.79	2.76 ± 0.75	0.015
HDL-C, mmol/L	1.56 ± 0.40	1.36 ± 0.34	<0.001

BAT, brown adipose tissue; SBP, systolic blood pressure; DBP, diastolic blood pressure; BMI; body mass index; ALT, alanine transaminase; AST, aspartate aminotransferase; UA, uric acid; BUN, blood urea nitrogen; Cr, Creatinine; FBG, fasting blood glucose; TG, triglyceride; TC, total cholesterol; LDL-C, low-density lipoprotein cholesterol; HDL-C, high-density lipoprotein cholesterol.

### High Levels of UA Inhibit BAT Thermogenic Capacity In Vitro

To determine the role of UA in the regulation of BAT function, we first examined the expression of *URAT1* in adipose tissue. As expected, the highest expression of *URAT1* was found in the kidney, while the levels in epididymal adipose tissue (eWAT) ranked second to the kidney. *URAT1* mRNA was also examined at relatively low levels in iWAT, BAT, and liver (Fig. 1*D*). To confirm whether extracellular UA could be transported into BAC through URAT1, primary BAT SVFs were induced to differentiate into mature brown adipocytes, which were then treated with UA and benzbromarone (a URAT1 inhibitor) (Fig. 1*E*). We detected the uptake of UA into BAC, which were treated with different concentrations (from 0 to 12 mg/dl) of UA for 24 h. Notably, the intracellular UA content increased significantly with increasing UA concentration (Fig. 1*F*), whereas it did not increase in the benzbromarone-treated group, suggesting that UA could indeed be transported into BAC. Meanwhile, we found that UA did not affect adipogenesis by oil red O staining and examining the expression of adipogenic genes (Supplemental Fig. S1, *G* and *H*). To better identify whether UA affects the thermogenic capacity of BAC, we treated the cells with CL-316,243, a selective β_3_-adrenergic receptor activator, to activate the thermogenic capacity of BAC (Fig. 1*G*), before examining thermogenic genes and oxygen consumption rate. We discovered a dose-dependent decline in the expression of thermogenic genes (Fig. 1*H*) and OCR (Fig. 1, *I* and *J*) of BAC. Next, we established the HUA model in BAC. The results showed that the downregulation of thermogenic genes and OCR by HUA in BAC could be reversed by benzbromarone (Fig. 1, *K*–*M*). Taken together, these results show that high levels of UA inhibit the thermogenic capacity of BAC in vitro.

### BAT Thermogenic Capacity Was Impaired in HUA Mice

**Figure 2.**
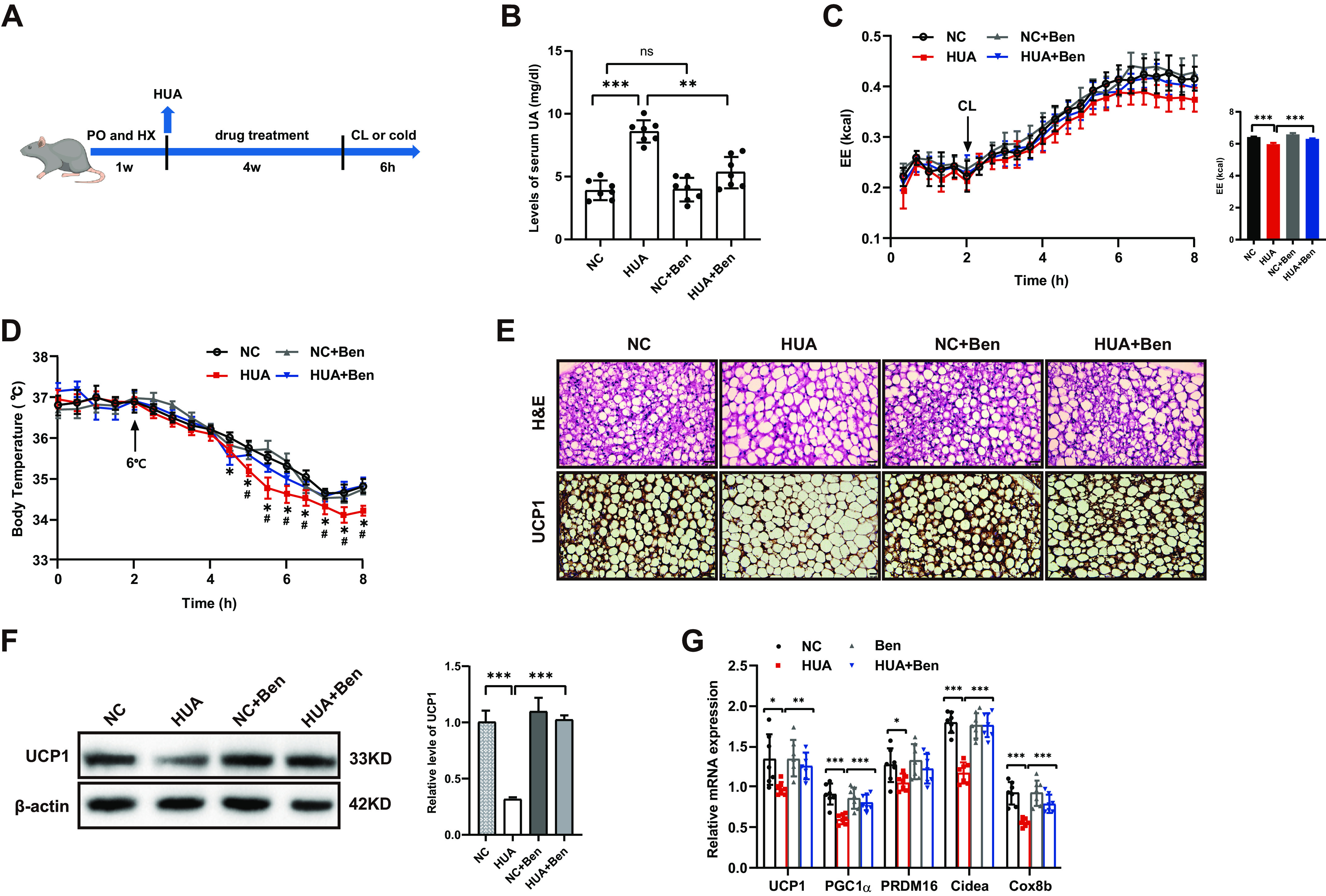
BAT thermogenic capacity was impaired in HUA mice. *A*: flowchart of the establishment of HUA mice and drug treatment, drug refers to benzbromarone (or metformin, AICAR and Compound C in the latter figure). To activate BAT, mice were either injected with CL-316,243 or placed in a cold (6°C) environment six hours before the experiments. *B*: serum UA levels of mice from different treatment groups (*n* = 7). *C*: energy expenditure (EE) in response to CL-316,243 injection determined by indirect calorimetry in mice from different treatment groups (*n* = 7). Bar diagram shows cumulative EE during 6 h after CL-316,243 treatment of the mice. *D*: core body temperature of mice from different treatment groups before and after transfer to cold (*n* = 7). *E*: H&E staining and protein expression of UCP1 detected by immunohistochemistry in the BAT of mice from different treatment groups (*n* = 7). Scale bar, 100 µm. *F*: protein expression of UCP1 in the BAT of mice from different treatment groups determined by Western blot (three independent experiments). *G*: mRNA expression of thermogenic genes in the BAT of mice from different treatment groups measured by qRT-PCR (*n* = 7). Data are shown as means ± SE. Statistical significance by one-way ANOVA (*P < 0.05, **P < 0.01, ***P < 0.001; (*D*) *HUA vs. NC, #HUA vs. HUA+Ben). *n* represents the number of mice/group. PO, potassium oxonate; HX, hypoxanthine; NC, normal control; HUA, hyperuricemia; Ben, benzbromarone treatment; UCP1, uncoupling protein1; PGC1α, peroxisome proliferator-activated receptor γ co-activator 1α; PRDM16, PR domain containing 16; Cidea, cell death-inducing DFFA-like effector A; Cox8b, cytochrome *c* oxidase 8b.

To provide further evidence for the role of UA in thermogenesis in vivo, we built HUA mice by intraperitoneal injection of PO and gavage of HX. Some of them were treated with benzbromarone to reduce serum UA and finally all mice were injected subcutaneously with CL-316,243 or placed in a cold (6°C) environment to activate the thermogenic activity of BAT (Fig. 2*A*). Compared to the control group, serum UA was significantly higher in HUA mice, whereas it decreased significantly after 4 wk of treatment with benzbromarone, a drug used to reduce serum UA by decreasing UA reabsorption in clinical practice (Fig. 2*B*). HUA mice exhibited similar body weight and adipose tissue weight (including WAT and BAT) to controls, and benzbromarone treatment did not affect body weight or adipose tissue weight (Supplemental Fig. S2, *A*–*C*). Consistent with the in vitro results, there was no change in the expression of adipogenic genes in BAT of HUA mice (Supplemental Fig. S2*D*). HUA mice showed less elevated energy expenditure during acute CL316,243 administration and lower body temperature in response to cold exposure, which could be alleviated by benzbromarone (Fig. 2, *C* and *D*), indicating that HUA suppresses BAT function. Compared to the control group, the lipid droplets of BAC were larger and the protein expression of UCP1 was downregulated, both of which could be reversed by benzbromarone (Fig. 2, *E* and *F*). In line with the in vitro results, the mRNA expression of thermogenic genes was also significantly reduced in HUA mice, which could also be reversed by benzbromarone (Fig. 2*G*). Collectively, these results demonstrate that the thermogenic capacity of BAT is impaired in HUA mice, implying that UA may regulate the process of thermogenesis.

### High Levels of UA Inhibit AMPK Phosphorylation in BAT

**Figure 3.**
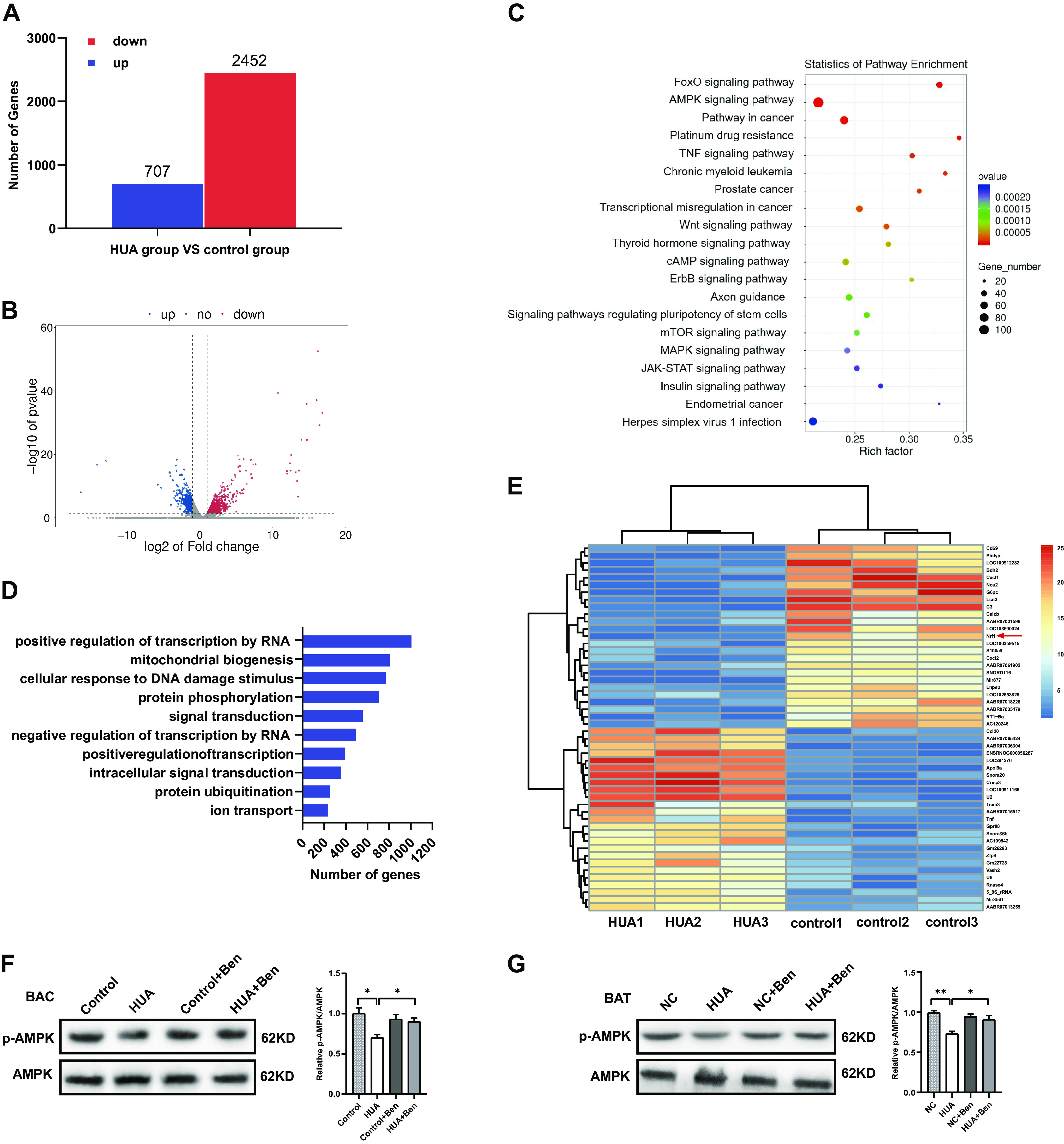
High levels of uric acid inhibit AMPK phosphorylation in BAT. RNA-Seq was performed on the primary BAC treated with or without 8 mg/dL UA for 24 h. *A*: column plot of differentially expressed genes in transcriptomics. *B*: volcano plot of differentially expressed genes in transcriptomics. *C*: KEGG analysis showed that “AMPK signaling pathway” was a major enriched alteration in complex cellular pathways. *D*: GO analysis showed that “mitochondrial biogenesis” was the second mainly enriched GO term. *E*: heat map screened “Nrf1” among the top 50 differentially expressed genes. *F*: phosphorylation of AMPK and total AMPK in the BAC treated with UA (8 mg/dL) and benzbromarone determined by Western blot (three independent experiments). The bar diagram shows the ratio of p-AMPK/AMPK. *G*: phosphorylation of AMPK and total AMPK in the BAT of mice from different treatment groups determined by Western blot (three independent experiments). The bar diagram shows the ratio of p-AMPK/AMPK. Data are shown as means ± SE. Statistical significance by Student’s *t* test or one way ANOVA (*P < 0.05, **P < 0.01). NC, normal control; HUA, hyperuricemia; Ben, benzbromarone; Nrf1: nuclear respiratory factor 1; AMPK, AMP-activated protein kinase.

To clarify the mechanism of UA in regulating thermogenesis, we performed RNA-seq on BAC treated with UA (8 mg/dl). A total of 3,159 differentially expressed genes were identified, including 2,452 downregulated and 707 upregulated genes (Fig. 3, *A* and *B*). KEGG analysis showed that the AMPK signaling pathway, which plays an important role in maintaining thermogenesis (23), was highly enriched with differentially expressed mRNAs (Fig. 3*C*). In addition, GO analysis revealed that “mitochondrial biogenesis,” which is regulated by the AMPK signaling pathway (24), was highly enriched with differentially expressed mRNAs (Fig. 3*D*). Furthermore, we screened nuclear respiratory factor 1 (*Nrf1*), a key transcription factor in the regulation of mitochondrial biogenesis (25), among the top 50 differentially expressed genes (Fig. 3*E*). Previous studies have shown that AMPK can enhance mitochondrial biogenesis by inducing *PGC1α* transcription, which activates a group of transcription factors, including *Nrf1* and *Nrf2*, which in turn activate mitochondrial transcription factor A (*Tfam*) (23, 25). Therefore, we speculated that HUA suppresses mitochondrial biogenesis by regulating AMPK, thereby inhibiting the thermogenic capacity of BAT. To confirm this hypothesis, we examined AMPK in BAC treated with high levels of UA (8 mg/dl) and found that UA suppressed AMPK phosphorylation (Thr172), whereas benzbromarone, which had no effect on AMPK phosphorylation itself but prevented BAC from taking up uric acid (Fig. 1*F*), reversed the HUA-induced inhibition of AMPK phosphorylation (Fig. 3*F*). Furthermore, the phosphorylation of AMPK was downregulated in the BAT of HUA mice, whereas benzbromarone which reduces serum UA in HUA mice (Fig. 2*B*), was able to rescue the downregulated phosphorylation of AMPK (Fig. 3*G*). These results indicate that HUA inhibits AMPK phosphorylation in BAT.

### Activation of AMPK Reverses UA-Induced Impairment of BAC Thermogenic Capacity In Vitro

**Figure 4.**
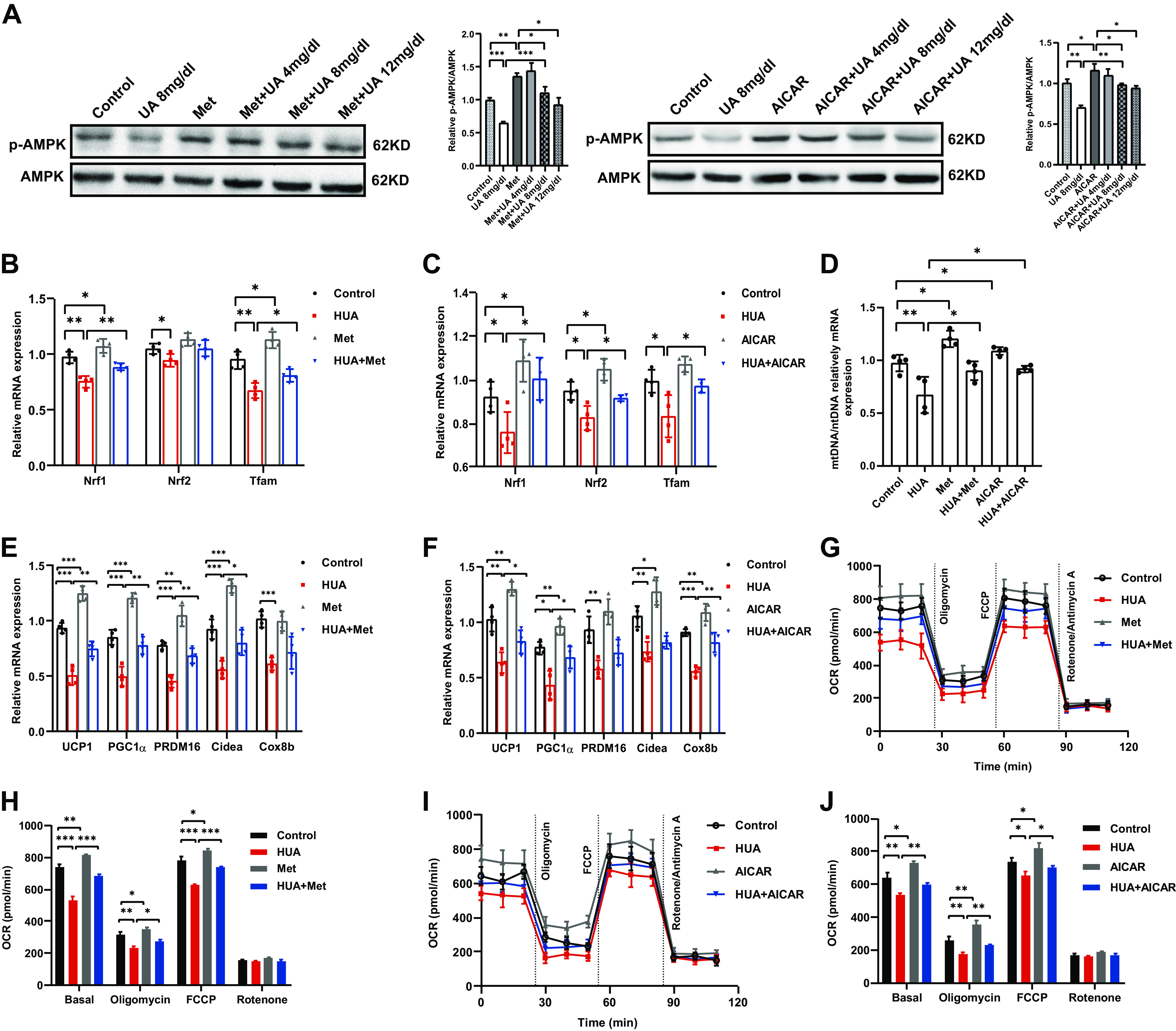
Activation of AMPK reverses UA-induced impairment of BAC thermogenic capacity in vitro. *A*: phosphorylation of AMPK and total AMPK in BAC from different treatment groups determined by Western blot (three independent experiments). The bar diagram shows the ratio of p-AMPK/AMPK. *B* and *C*: mRNA expression of mitochondrial biogenesis related genes in the BAC of different treatment groups measured by qRT-PCR (*n* = 4). *D*: mitochondrial content of the BAC from different treatment groups measured by qRT-PCR (*n* = 4). *E* and *F*: mRNA expression of thermogenic genes in BAC from different treatment groups measured by qRT-PCR (*n* = 4). *G*: oxygen consumption rate (OCR) of the BAC from different treatment groups measured with an XF24 extracellular flux analyzer (*n* = 4). *H*: the average OCR of the BAC described in (*G*) at different stages. *I*: oxygen consumption rate (OCR) of the BAC from different treatment groups measured with an XF24 extracellular flux analyzer (*n* = 4). *J*: the average OCR of the BAC described in (*I*) at different stages. Data are shown as means ± SE. Statistical significance by one way ANOVA (*P < 0.05, **P < 0.01, ***P < 0.001). *n* represents the number of cell samples/group. UA, uric acid; Met, metformin; AICAR, 5-aminoimidazole-4-carboxamide ribonucleotide; HUA, hyperuricemia; AMPK, AMP-activated protein kinase; Nrf, nuclear respiratory factor; Tfam, mitochondrial transcription factor A; UCP1, uncoupling protein1; PGC1α, peroxisome proliferator-activated receptor γ co-activator 1α; PRDM16, PR domain containing 16; Cidea, cell death-inducing D FFA-like effector A; Cox8b, cytochrome *c* oxidase 8b; FCCP, carbonyl cyanide 4-trifluoromethoxy phenylhydrazone.

To further clarify the inhibition of AMPK phosphorylation by UA in BAT, we added the AMPK activators metformin or AICAR to the UA-treated BAC. We found that AMPK activators alleviated the inhibition of AMPK phosphorylation by UA. What's more, UA attenuated the activation of AMPK by Met or AICAR in a dose-dependent manner (Fig. 4*A*). Consistent with RNA sequencing, our results showed that UA suppressed the expression of *Nrf1*, *Nfr2* and *Tfam*, key transcription factors regulating mitochondrial biogenesis (Figs. 4*B* and 4C). Furthermore, UA reduced the mitochondrial content in BAC (Fig. 4*D*). Activation of AMPK could reverse the inhibitory effect of UA described above. These results suggest that UA may inhibit mitochondrial biogenesis via AMPK, which is an important part of thermogenesis. We then tested whether activation of AMPK could reverse the impaired thermogenic capacity of BAC induced by UA in vitro. The results showed that metformin and AICAR could ameliorate the inhibition of thermogenic gene expression (Fig. 4, *E* and *F*) and oxygen consumption rate (Fig. 4, *G*–*J*) by UA.

### Activation of AMPK Reverses the Impairment of BAT Thermogenic Capacity in HUA Mice

**Figure 5.**
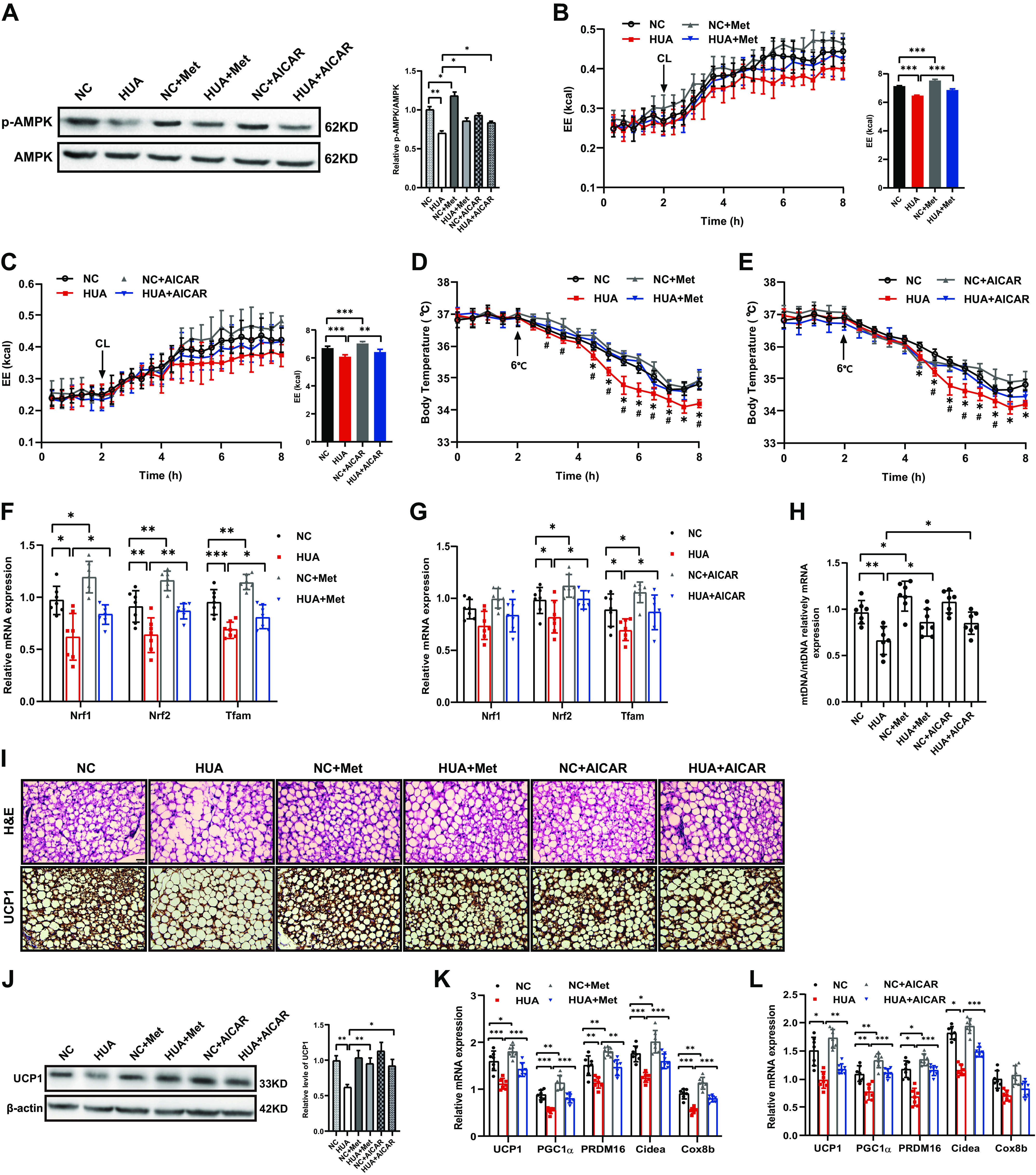
Activation of AMPK reverses the impairment of BAT thermogenic capacity in HUA mice. *A*: Phosphorylation of AMPK and total AMPK in the BAT of mice from different treatment groups determined by Western blot (three independent experiments). Bar diagram shows the ratio of p-AMPK/AMPK. *B* and *C*: energy expenditure (EE) in response to CL-316,243 injection determined by indirect calorimetry in mice from different treatment groups (*n* = 7). The bar diagram shows cumulative EE during 6 h after CL-316,243 treatment of the mice. *D* and *E*: core body temperature of mice from different treatment groups before and after transfer to cold (*n* = 7). *F* and *G*: mRNA expression of mitochondrial biogenesis related genes in the BAT of mice from different treatment groups measured by qRT-PCR (*n* = 7). *H*: mitochondrial content of the BAT in mice from different treatment groups measured by qRT-PCR (*n* = 7). *I*: H&E staining and protein expression of UCP1 detected by immunohistochemistry in the BAT of mice from different treatment groups (*n* = 7). Scale bar, 100 µm. *J*: protein expression of UCP1 in the BAT of mice from different treatment groups determined by Western blot (three independent experiments). *K* and *L*: mRNA expression of thermogenic genes in the BAT of mice from different treatment groups measured by qRT-PCR (*n* = 7). Note: Figs. 5*D* and 5*E* were conducted in the same experiment as Fig. 2*D* and the three figures shared the same NC and HUA groups, we just presented them separately. Data are shown as means ± SE. Statistical significance by one-way ANOVA (*P < 0.05, **P < 0.01, ***P < 0.001; (*D*) and (*E*) *HUA vs. NC, #HUA vs. HUA+Met/AICAR). *n* represents the number of mice/group. NC, normal control; Met, metformin; AICAR, 5-aminoimidazole-4-carboxamide ribonucleotide; HUA, hyperuricemia; AMPK, AMP-activated protein kinase; Nrf, nuclear respiratory factor; Tfam, mitochondrial transcription factor A; UCP1, uncoupling protein1; PGC1α, peroxisome proliferator-activated receptor γ co-activator 1α; PRDM16, PR domain containing 16; Cidea, cell death-inducing D FFA-like effector A; Cox8b, cytochrome *c* oxidase 8b.

To investigate whether activation of AMPK can reverse BAT thermogenic capacity in vivo, HUA mice were administrated with two AMPK activators: metformin or AICAR. Activation of AMPK had no effect on the serum UA concentration, nor on the changes in body weight and adipose tissue weight (Supplemental Fig. S2, *E*–*I*). As expected, we found that AMPK activators ameliorated the reduced p-AMPK in HUA mice (Fig. 5*A*). Energy expenditure of HUA mice was also significantly increased by metformin and AICAR (Fig. 5, *B* and *C*), as well as body temperature(Fig. 5, *D* and *E*). Consistent with the in vitro results, the expression of *Nrf1*, *Nfr2* and *Tfam* (Figs. 5, *F* and *G*) and mitochondrial content (Fig. 5*H*) were significantly lower in the BAT of HUA mice, which could be reversed by metformin or AICAR. In addition, activation of AMPK in HUA mice could also restore lipid droplet size (Fig. 5*I*), UCP1 protein expression (Fig. 5*J*), and thermogenic gene mRNA expression (Fig. 5, *K* and *L*). The results demonstrate that activation of AMPK can reverse the impairment of BAT thermogenic capacity in HUA mice.

### Inhibition of AMPK Aggravates the Inhibitory Effect of HUA on BAC Thermogenic Capacity In Vitro

**Figure 6.**
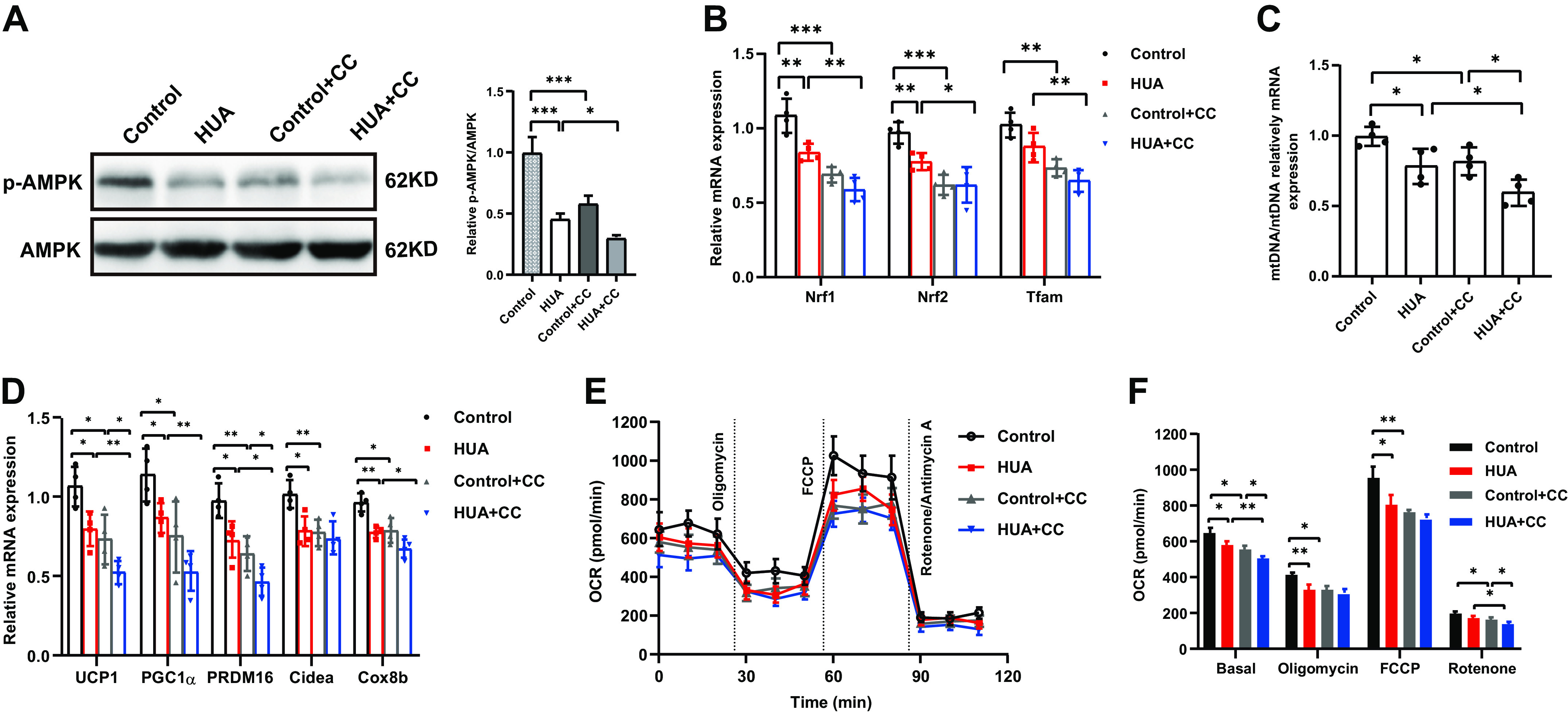
Inhibition of AMPK aggravates the inhibitory effect of HUA on BAC thermogenic capacity in vitro. *A*: phosphorylation of AMPK and total AMPK in BAC from different treatment groups determined by Western blot (three independent experiments). The bar diagram shows the ratio of p-AMPK/AMPK. *B*: mRNA expression of mitochondrial biogenesis related genes in the BAC of different treatment groups measured by qRT-PCR (*n* = 4). *C*: mitochondrial content of the BAC from different treatment groups measured by qRT-PCR (*n* = 4). *D*: mRNA expression of thermogenic genes in BAC from different treatment groups measured by qRT-PCR (*n* = 4). *E*: oxygen consumption rate (OCR) of the BAC from different treatment groups measured with an XF24 extracellular flux analyzer (*n* = 4). *F*: the average OCR of the BAC described in (*E*) at different stages. Data are shown as means ± SE. Statistical significance by one way ANOVA (*P < 0.05, **P < 0.01, ***P < 0.001). *n* represents the number of cell samples/group. HUA, hyperuricemia; CC, Compound C; AMPK, AMP-activated protein kinase; Nrf, nuclear respiratory factor; Tfam, mitochondrial transcription factor A; UCP1, uncoupling protein1; PGC1α, peroxisome proliferator-activated receptor γ co-activator 1α; PRDM16, PR domain containing 16; Cidea, cell death-inducing D FFA-like effector A; Cox8b, cytochrome *c* oxidase 8b; FCCP, carbonyl cyanide 4-trifluoromethoxy phenylhydrazone.

To provide further evidence that HUA inhibits AMPK phosphorylation in BAC, we treated BAC with CC (Fig. 6*A*), an AMPK inhibitor. As previous studies have shown that AMPK is a key regulator of BAT thermogenesis, CC exhibited the same inhibitory effect on thermogenesis as HUA in vitro. CC suppressed the expression of *Nrf1*, *Nfr2*, and *Tfam* (Fig. 6*B*), reduced mitochondrial content (Fig. 6*C*), and downregulated thermogenic genes (Fig. 6*D*) and OCR (Fig. 6, *E* and *F*). However, CC aggravated the inhibitory effect of HUA on the thermogenic capacity of BAC.

### Inhibition of AMPK Exacerbates the Impairment of BAT Thermogenic Capacity in HUA Mice

**Figure 7.**
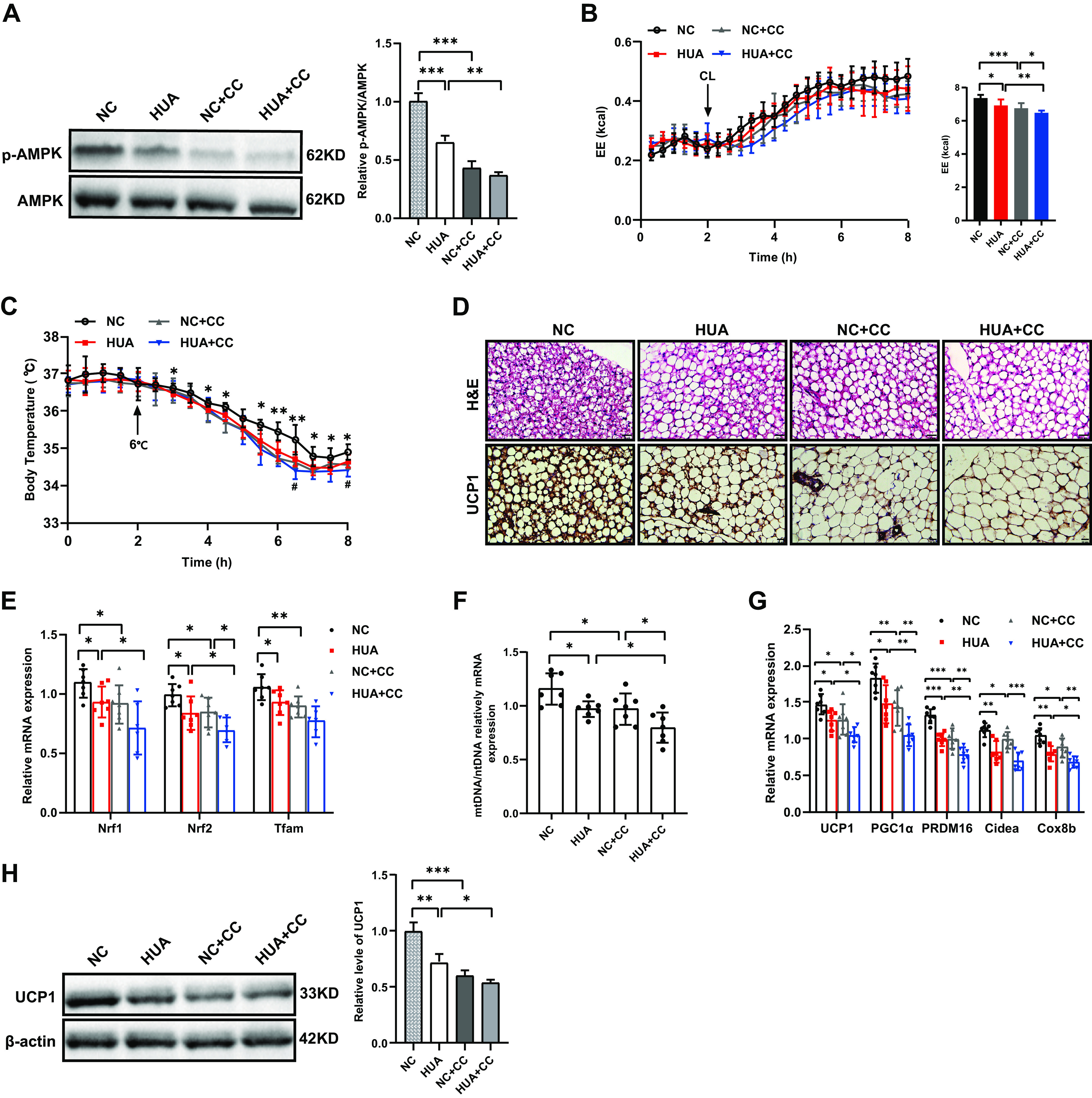
Inhibition of AMPK exacerbates the impairment of BAT thermogenic capacity in HUA mice. *A*: phosphorylation of AMPK and total AMPK in the BAT of mice from different treatment groups determined by Western blot (three independent experiments). Bar diagram shows the ratio of p-AMPK/AMPK. *B*: energy expenditure (EE) in response to CL-316,243 injection determined by indirect calorimetry in mice from different treatment groups (*n* = 7). The bar diagram shows cumulative EE during 6 h after CL-316,243 treatment of the mice. *C*: core body temperature of mice from different treatment groups before and after transfer to cold (*n* = 7). *D*: H&E staining and protein expression of UCP1 detected by immunohistochemistry in the BAT of mice from different treatment groups. (*n* = 7). Scale bar, 100 µm. *E*: mRNA expression of mitochondrial biogenesis related genes in the BAT of mice from different treatment groups measured by qRT-PCR (*n* = 7). *F*: mitochondrial content of the BAT in mice from different treatment groups measured by qRT-PCR (*n*=7). *G*: mRNA expression of thermogenic genes in the BAT of mice from different treatment groups measured by qRT-PCR (*n* = 7). *H*: protein expression of UCP1 in the BAT of mice from different treatment groups determined by Western blot (three independent experiments). Data are shown as means ± SE. Statistical significance by one-way ANOVA (*P < 0.05, **P < 0.01, ***P < 0.001; *HUA vs. NC, #*HUA* vs. HUA+CC). *n* represents the number of mice/group. NC, normal control; CC, Compound C; HUA, hyperuricemia; AMPK, AMP-activated protein kinase; Nrf, nuclear respiratory factor; Tfam, mitochondrial transcription factor A; UCP1, uncoupling protein1; PGC1α, peroxisome proliferator-activated receptor γ co-activator 1α; PRDM16, PR domain containing 16; Cidea, cell death-inducing D FFA-like effector A; Cox8b, cytochrome *c* oxidase 8b.

Similarly, we also test the effect of AMPK inhibitor on HUA mice. Inhibition of AMPK had no effect on the serum UA concentration nor on the changes in body weight and adipose tissue weight (Supplemental Fig. S2, *J*–*M*). As expected, CC showed the same inhibitory effect on thermogenesis as HUA mice (Fig. 7*A*), with lower energy expenditure (Fig. 7*B*), lower body temperature (Fig. 7*C*), and larger lipid droplets (Fig. 7*D*) than normal control. In addition, CC also suppressed the expression of *Nrf1*, *Nfr2*, and *Tfam* (Fig. 7*E*), reduced mitochondrial content (Fig. 7*F*), and downregulated thermogenic genes (Fig. 7, *G* and *H*) of BAT. However, CC exacerbates the impairment of BAT thermogenic capacity in HUA mice. Taken together, the results of AMPK inhibitor in vitro and in vivo suggest that HUA may also affect other signaling pathways besides AMPK signaling. Therefore, HUA may inhibit the thermogenic capacity of BAT through both AMPK-dependent and independent mechanisms.

## DISCUSSION

In this study, we identified UA as a novel regulator of thermogenesis. First, in vitro experiments showed that high levels of UA suppressed thermogenic gene expression and oxygen consumption rate in BAC. Accordingly, in vivo results showed that HUA mice exhibited lower energy expenditure and body temperature, with larger lipid droplets and lower thermogenic gene expression in BAT. Finally, all of the above inhibitory effects of HUA were reversed by benzbromarone. Taken together, our findings demonstrate for the first time that UA inhibits BAT thermogenic capacity.

UA is mainly transported by URAT1, as it exists as an anion and cannot cross membranes by itself (26). Both previous studies and our research have shown the existence of URAT1 in adipocytes, and that extracellular UA can be transported into adipocytes through URAT1 (5, 16). Recent studies suggest that UA suppresses the expression of *UCP1*, *PRDM16*, and *PGC1α* in 3T3-L1 adipocytes (4). In our study, we found that UA also inhibited these transcription factors in primary brown adipocytes, ultimately inhibiting of thermogenesis. However, the exact effects of UA on BAT thermogenic capacity and the underlying molecular mechanisms are poorly understood.

To further elucidate the mechanism of UA on brown adipocytes, mRNA-sequencing analysis was performed. KEGG enrichment pathway and GO analysis indicated that “AMPK signaling pathway” and “mitochondrial biogenesis” were highly enriched with differentially expressed mRNAs. Therefore, we speculated that high levels of UA inhibit mitochondrial biogenesis by regulating AMPK signaling pathway, thereby inhibiting BAT thermogenic capacity, and our results confirmed this hypothesis. First, the phosphorylation of AMPK was suppressed by HUA, and UA attenuated the activation of AMPK by Met or AICAR in a dose-dependent manner. Second, the expression of *Nrf1*, *Nfr2*, and *Tfam* and the mitochondrial content were inhibited by HUA. Third, activation of AMPK alleviated the inhibition of AMPK phosphorylation by HUA and increased mitochondrial biogenesis, thereby restoring impaired BAT thermogenic capacity in vitro and vivo.

AMPK is a highly conserved protein composed of catalytic α (α1, α2), and regulatory β (β1, β2) and γ (γ1, γ2, γ3) subunits (23). Numerous studies have demonstrated that AMPK is essential for brown adipose function by ablating of AMPK in adipose tissue. The adipose-specific AMPK null mice had a profound defect in thermogenesis, with a rapid drop in core body temperature compared to controls when housed in a cold environment (27, 28). Consistent with these findings, our study found that activation of AMPK by metformin or AICAR could restore the impairment of BAT thermogenic capacity induced by HUA. Furthermore, mitochondrial function was impaired in AMPKβ AKO mice (28). AMPK not only regulates mitochondrial biogenesis through communication with the transcriptional co-activator PGC1α and transcription factor EB (TFEB), but also controls fission through mitochondrial fission factor (MFF), and mitophagy through ULK1 (29). Mitochondria play a key role in the process of BAT thermogenic capacity. PGC-1α plays a central role in a regulatory network governing the transcriptional control of mitochondrial biogenesis and respiratory function by targeting several transcription factors, including Nrf1 and Nrf2 (25). Therefore, AMPK enhances mitochondrial biogenesis by inducing PGC-1α transcription, which activates Nrf1 and Nrf2, which in turn activate mtTFA. Thus, AMPK is essential for maintaining BAT mitochondrial content and function (23). In our study, HUA suppressed the mitochondrial content and the expression of *Nrf1*, *Nfr2*, and *Tfam*, which could be restored by AMPK activators, subsequently restoring mitochondrial respiration. However, the potential mechanism by which UA inhibits AMPK in BAT is unclear. Prior studies have provided important information about upstream activators of AMPK, namely metabolic stress, liver kinase B1 (LKB1), CaM-dependent protein kinase kinase (CaMKK), and hormones (30). Perhaps UA inhibits these upstream activators of AMPK, but this has not been previously reported and needs to be confirmed in our next studies. In addition, we found that inhibition of AMPK aggravated the inhibitory effect of HUA on BAT thermogenic capacity, suggesting that HUA may also affect other signaling pathways besides AMPK signaling. Therefore, HUA may inhibit the thermogenic capacity of BAT through both AMPK-dependent and independent mechanisms.

### Conclusions

In summary, we identified a novel negative regulator of BAT thermogenic capacity. Furthermore, we unveiled that the inhibitory effect of UA on BAT thermogenesis may be mediated by AMPK. Finally, our study uncovers a novel mechanism underlying the regulation of brown adipose tissue function, providing a new strategy to combat obesity.

## DATA AVAILABILITY

Data will be made available upon reasonable request.

## SUPPLEMENTAL DATA

10.6084/m9.figshare.23821008 Supplemental Figs. S1 and S2: https://doi.org/10.6084/m9.figshare.23821008.

10.6084/m9.figshare.23821143 Supplemental Tables S1–S3: https://doi.org/10.6084/m9.figshare.23821143.

10.6084/m9.figshare.23821170 Supplemental Methods: https://doi.org/10.6084/m9.figshare.23821170.

## GRANTS

This work was supported by grants from the Science and Technology Development Fund of Nanjing Medical University (NMUB2020152), and the Natural Science Foundation of Jiangsu Province (BK20140459).

## DISCLOSURES

No conflicts of interest, financial or otherwise, are declared by the authors.

## AUTHOR CONTRIBUTIONS

M.D., K.A., and L.M. conceived and designed research; M.D. and K.A. performed experiments; M.D. and K.A. analyzed data; M.D., K.A., and L.M. interpreted results of experiments; M.D. prepared figures; M.D., K.A., and L.M. drafted manuscript; M.D., K.A., and L.M. edited and revised manuscript; M.D., K.A., and L.M. approved final version of manuscript.
